# CYP1A2 polymorphism may contribute to agomelatine-induced acute liver injury: Case report and review of the literature

**DOI:** 10.1097/MD.0000000000027736

**Published:** 2021-11-12

**Authors:** Shushan Wang, Qing Xu, Kankan Qu, Jun Wang, Zhenhe Zhou

**Affiliations:** a The Affiliated Wuxi Mental Health Center of Nanjing Medical University, Department of Pharmacy, Binhu District, Wuxi City, Jiangsu Province, China; b The Affiliated Wuxi Mental Health Center of Nanjing Medical University, Department of Psychiatry, Binhu District, Wuxi City, Jiangsu Province, China.

**Keywords:** agomelatine, case report, cytochrome P450 family 1 subfamily A member 2, hepatotoxicity, metabolite

## Abstract

**Rationale:**

Liver function monitoring is recommended when agomelatine is prescribed, although liver enzymes are not considered predictive biomarkers. Most patients present with acute liver injury, with only a few presenting with levels of liver enzymes that are over 30 times the upper limit of normal. The patient-specific risk factors that are associated with liver injury remain unclear. Thus, this report provides new insights into the mechanism of agomelatine-induced acute hepatocellular injury based on cytochrome P450 family 1 subfamily A member 2 (CYP1A2) polymorphism.

**Patient concerns:**

We present a case of acute hepatocellular injury in a 75-year-old man who was taking agomelatine at a dose of 50 mg/qn. All hepatitis virus test results were negative. No history of liver disease was observed. As CYP1A2 is the main metabolic enzyme of agomelatine, CYP1A2 AA (rs762551) genetic polymorphism was analyzed.

**Diagnosis:**

The patient's transaminases level exceeded the critical value on day 72 after starting oral agomelatine.

**Interventions:**

The patient received intravenous magnesium isoglycyrrhizinate, a liver cell-protecting agent, followed by the withdrawal of agomelatine.

**Outcomes:**

There was an improvement in the levels of the liver enzymes and no subsequent organ dysfunction was observed.

**Lessons:**

Here, we report a case of acute hepatocellular injury characterized by a very high aspartate aminotransferase level. Periodic liver function testing throughout the treatment period can help in the rapid and appropriate diagnosis of acute liver injury, particularly in the absence of typical clinical manifestations. Agomelatine hepatic toxicity might be related to an idiosyncratic metabolic reaction that depends on individual patient differences. As it is the main metabolic enzyme of agomelatine, CYP1A2 genetic polymorphism may contribute to liver injury by affecting its metabolites.

## 1 Introduction

Agomelatine (AGM) is a melatonin analogue with agonistic properties. Moreover, it antagonizes serotonin 5-hydroxytryptamine (2C) receptors without affecting the neuronal uptake of monoamines.^[1]^ It has been licensed for the treatment of major depressive disorder in adults since 2009 in the European Union, since 2010 in Australia, and since 2011 in China. After the widespread prescription of AGM, there is mounting evidence that liver injury is associated with the use of AGM.^[2,3]^ Therefore, the European Medicines Agency identified AGM-associated “hepatotoxic reactions” as a new safety concern in September 2013. In October 2013, the manufacturer of AGM responded to these concerns by releasing safety information letters and providing guidance for the continuous monitoring of liver function by the psychiatrists prescribing AGM. Considering these aspects, it seems inappropriate to evaluate AGM as an antidepressant agent for the first choice.^[4]^ These measures may have induced selective prescription, and could explain why AGM is not associated with an increased risk of hospitalization for liver injury. Therefore, the low risk of liver injury with AGM should be interpreted in the context of the European risk minimization measures.^[5]^

It has been estimated that age >50 years, female sex, polypharmacy, and liver disease might be risk factors for the development of AGM-induced hepatotoxicity.^[6–8]^ However, no specific patient population has been identified with the potential risk factors.^[9]^ Furthermore, the underlying mechanism of acute liver injury appears to be idiosyncratic and depends on individual patient differences. AGM is rapidly absorbed from the gastrointestinal tract and immediately transported to the liver,^[10]^ where 90% of it is metabolized by cytochrome P450 isoenzymes. Subsequently, 7-desmethyl-, 3-hydroxy-, and 3-hydroxy-7-desmethyl-AGM were identified as the 3 metabolites of AGM and showed less activity than the parent drug.^[11]^ It has been observed that the bioactivation of AGM leads to the formation of epoxides. These reactive metabolites may be closely related to AGM toxicity.^[12]^ Cytochrome P450 family 1 subfamily A member 2 (CYP1A2) activity has been directly correlated with AGM metabolism.^[13]^ Many variants of CYP1A2 have been reported, with some having an impact on drug metabolism. Among single nucleotide polymorphism (SNP), SNP rs762551 is the most well-studied genetic variant of CYP1A2. Individuals with the rs762551AA genotype are considered to have increased metabolism compared to those with the AC and CC genotypes.^[14]^

Since the marketing of AGM, cases have been reported with liver enzyme elevations of >10 × upper limit of normal (ULN) as rare side effects. In this report, we have described the case of a male patient with AGM-induced liver enzyme elevation >30 × ULN. Furthermore, we have examined the pharmacogenetic testing of the patient and explored some possible molecular mechanisms from the literature.

## 2 Case presentation

A 75-year-old man had a history of recurrent depressive disorder. At the age of 40 years, he was first diagnosed with a major depressive episode of moderate intensity, characterized by low mood and anhedonia. During the past decades, he had been treated with a variety of antidepressants with a single or combined treatment strategy as well as 14 times with modified electric convulsive therapy. The treatment strategies included the following drugs singly or in combination: chlorpromazine 75 mg, amitriptyline 100 mg, trazodone 150 mg, fluvoxamine 200 mg, fluoxetine, paroxetine, duloxetine, escitalopram, sertraline, venlafaxine 225 mg, mirtazapine 45 mg, and olanzapine, and only short-term effects were observed. However, the patient's condition often fluctuated with recurrence of depression.

In the current depressive episode, he was prescribed AGM for the first time because of its unique pharmacological mechanism. Liver function was analyzed before and after AGM intake, according to the manufacturer's recommendations. Examination of the electronic medical records of our hospital revealed that he never had any liver disease or abnormal liver function. Furthermore, there was no history of alcohol consumption, and tests for hepatitis A, hepatitis B, and hepatitis C viruses were all negative.

He had been diagnosed with type 2 diabetes mellitus and hypertension for at least 10 years, and was taking metoprolol 25 mg bid, acarbose 50 mg qd, and protamine biosynthesis human insulin 15 u qd subcutaneous injection. His fasting blood glucose was 6.49 mmol/L (3.9–6.1 mmol/L), blood pressure 146/85 mm Hg, and heart rate 86 beat per minute.

Considering the age of the patient and associated diseases, there was a potentially high risk of side effects, but the psychiatrists were hopeful that the new pharmacological mechanism of AGM could provide some benefits. Moreover, in 2016, it was reported that a 91-year old patient took AGM (25 mg/day), which relieved his symptoms without any adverse effects.^[15]^ A two-arm, single-blind randomized controlled trial recruited patients with type 2 diabetes mellitus between the ages of 18 to 80 years who were treated with AGM 25 to 50 mg/day. AGM was more effective and acceptable than fluoxetine, and no liver injury was observed.^[16]^ In this patient, the prescribed first dose was 25 mg qn, which was increased to 50 mg qn on the 21st day (day 21). The titration dose was increased cautiously and slowly.^[17]^

## 3 Liver function monitoring

**Figure 1:**
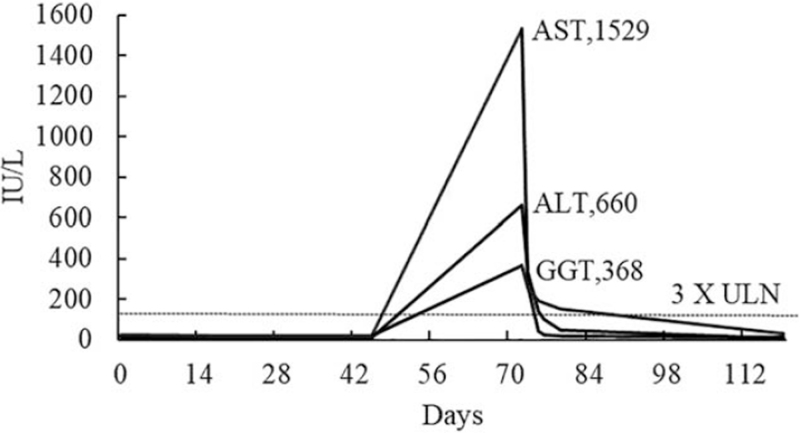
Line chart showing the increase in the levels of selected liver enzymes in tests conducted on different days from the start of AGM therapy. AST value peaked at 1529 IU/L, above 3 × ULN; ALT value peaked at 660 IU/L, above 13 × ULN; GGT value peaked at 368 IU/L, above 3 × ULN; normal values in our institution: AST 5 to 50 IU/L; ALT 8 to 50 IU/L; GGT 7 to 50 IU/L. ULN = upper limit of normal, AST = aspartate aminotransferase, ALT = alanine aminotransferase, GGT = γ-glutamyltranspeptidase.

**Figure 2:**
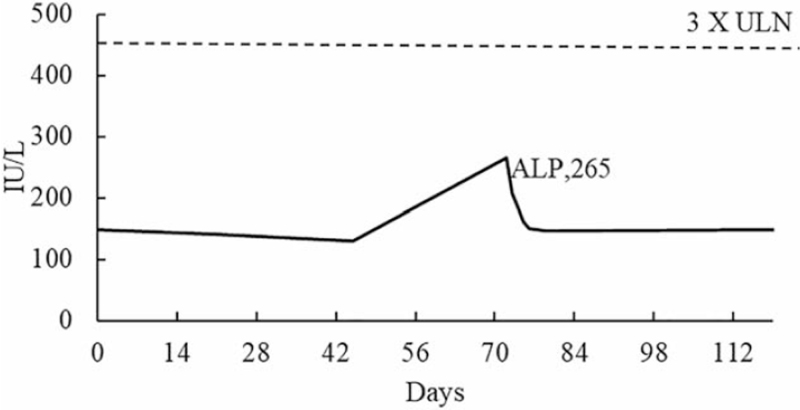
Line chart showing the increase in the ALP levels in tests conducted on different days from the start of AGM therapy. The ALP level steadily increased to a maximum of 265 IU/L. Normal value at our institution: ALP 40 to 150 IU/L. ULN = upper limit of normal, ALP = alkaline phosphatase.

Liver function was monitored at baseline (day 0), and in week 3 (day 21) and week 6 (day 45). All results were within the normal range, as shown in Figures 1 and 2. As the treatment progressed, the patient's symptoms gradually improved, with no discomfort or abnormal signs. Liver enzymes were checked during week 11 (day 72), and showed a sharp elevation with aspartate aminotransferase (AST) and alanine aminotransferase (ALT) levels rising to above 1529 IU/L (>30 × ULN) and 660 IU/L (>13 × ULN) (Fig. 1), respectively. The γ-glutamyltranspeptidase level was also above 3 × ULN. However, the alkaline phosphatase (ALP) level was only above 1 × ULN, as shown in Figure 2.

## 4 Outcomes: treatment after the elevation of liver enzymes and genetic testing

After receiving a telephone call from the laboratory that the AST and ALT levels exceeded the alert value, we immediately rechecked the liver function and stopped AGM administration that night. However, we did not change the treatment for somatic diseases. The laboratory examination results supported acute liver injury. However, they are not shown in the figures because the time of blood collection was random, and the existing results as well as the subsequent laboratory examination results were all evaluated at a fixed time point of 6:30 a.m.

The patient was treated with intravenous (IV) 0.9% sodium chloride (250 mL) and IV magnesium isoglycyrrhizinate 200 mg qd, which is a liver cell-protecting agent with cortisone analogues extracted from licorice plants. During the next week, the patient agreed to undergo treatments that led to additional hospitalization expenses of over 500 RMB. We continued to monitor liver function on days 73, 75, 76, and 79. We found that 3 of the liver enzymes decreased to normal levels within a week. Only the γ-glutamyltranspeptidase levels returned to normal on day 119. The patient had no obvious clinical features from the beginning to the end of the study. There were no significant fluctuations in heart rate, blood pressure, and blood glucose levels, and no abnormalities in the electrocardiogram or myocardial enzyme spectrum. Abdominal ultrasonography and computed tomography findings were normal. The Roussel Uclaf causality assessment method indicated a probable adverse reaction. As AGM-related hepatotoxicity is mostly hepatocellular, there is no doubt that the diagnosis of liver injury caused by AGM is accurate.

At a follow-up visit (day 119), the patient was informed that we aimed to evaluate the effects of genetic polymorphism on the metabolizing enzymes. The patient agreed to undergo a fluorescence polymerase chain reaction test to detect CYP1A2 SNPs. Genetic testing results indicated that the patient had the rs762551 AA genotype with enhanced CYP1A2 metabolism. We obtained informed consent from the patient for publication of this case report.

## 5 Discussion

### 5.1 Pathophysiological types of drug-induced liver injury

AGM increases liver weight and coefficient, and causes pathological liver injury.^[18]^ AGM-related hepatotoxicity is mostly hepatocellular, and cholestatic or hypersensitivity reactions have not yet been reported.^[19]^ In Roussel Uclaf causality assessment, the R-ratio is often used to distinguish between different patterns of liver injury in patients with liver injury. The R-ratio can be calculated as follows: ALT value/ALT upper limit of normal versus the ALP value/ALP upper limit of normal. An R-ratio >5 indicated a hepatocellular injury pattern. In our patient, the R-ratio was greater than 7.4. A predominant increase in transaminase activity reflects a disturbance in hepatocellular integrity. However, liver enzymes are neither sensitive nor specific for predicting the severity of hepatotoxicity.^[20,21]^ Although the AST level in this patient was up to 30 times of the ULN, there were hardly any clinical manifestations. This reflects the powerful compensatory ability of the liver and also increases the likelihood of missing the diagnosis in the absence of liver function monitoring. In addition, we found evidence of liver injury during routine liver function examination on the 72nd day after starting the medication. This shows the necessity of performing liver function tests, even if the drug has been used for more than 2 months.

### 5.2 Drug metabolism

Using ultra-high-performance liquid chromatography and quadrupole time-of-flight mass spectrometry analyses, 38 AGM metabolites and adducts were identified, of which 32 were reactive metabolites.^[22]^ These reactive metabolites play a critical role in the pathogenesis of idiosyncratic adverse drug reactions. Excess reactive electrophiles can covalently bind to proteins, deoxyribonucleic acid molecules, and other biomolecules. It is believed that in certain cases, cellular function is compromised and organ toxicity can occur. In a study on human liver microsomes, 5 reduced glutathione-trapped adducts and 2 semicarbazide-trapped aldehydes were uncovered.^[12]^ These reactive metabolites have a certain incubation period for the consumption of reducing substances in vivo, which is also related to the susceptibility of patients. There is considerable variability in intra- and inter-individual bioavailability.^[23]^ It is not clear if this is responsible for the complexity of the idiosyncratic clinical observations. Some studies have demonstrated epoxide metabolites, which could be utilized to further understand the mechanism of adverse effects related to AGM.^[24,25]^

### 5.3 CYP1A2 activity

CYP1A2 is the primary enzyme that contributes to the formation of AGM-reduced glutathione adducts and AGM-hydrazones. CYP1A2 activity shows pronounced intra- and inter-individual variability based on the polymorphism of inducers, but not of inhibitors.^[26]^ CYP1A2 inducers augment the formation of reactive metabolites, which may increase AGM-related toxicity. It has been clinically demonstrated that smoking induces CYP1A2 and decreases the bioavailability of AGM. Although AGM already has a low bioavailability, an inducer may further decrease its bioavailability by accelerating its metabolism. These mechanisms are compatible with the hypothesis that genetic variability in drug metabolism or bioactivation is a major determinant of acute liver injury.^[27]^

### 5.4 Pharmacogenetics

Several studies have reported the influence of CYP1A2 rs762551 in patients with the A allele, leading to lower AGM serum drug concentrations and higher reactive metabolite concentrations, while it leads to higher plasma drug levels in patients with the C allele.^[28]^ The AA genotype is considered a “fast” AGM metabolizer while the C allele is "slow or poor,” as shown in Table 1.

**Table 1:** Effects of CYP1A2 rs762551 polymorphism on enzyme activity characterized by AGM AUC and Cmax.

Genotype	Total volunteers	AGM Dose	CYP1A2 activity	Study
∗1FFand BB	Total 28 healthy, non-smoker, no intake of coffee or tea	25 mg oral	Lower AGM concentration	Saiz-Rodríguez M 2019^[13]^
rs762551 AA	Total 72 healthy, Chinese non-smoker, no intake of coffee or tea	25 mg oral	Lower Level of AGM exposure (AUC, Cmax)	L Song, 2014^[28]^
rs762551 AA	Total 3570 individuals, systematic review and meta-analysis		Lower level of AGM exposure (AUC, Cmax)	Koonrungsesomboon N 2018^[29]^
rs762551 CC	72 healthy, Chinese non-smoker, no intake of coffee or tea	25 mg oral	Much higher mean of AGM AUC and Cmax	L Song 2014^[28]^

Some studies have reported results for CYP1A2∗1F but only measured CYP1A2: (−163) C>A (rs762551). AUC = area under the time to concentration curve, Cmax = maximal concentration.

The findings of a study involving 3570 individual participants indicated the functional impact of rs762551 polymorphism on CYP1A2 variation in humans.^[29]^ Higher enzyme activity and lower levels of AGM exposure were observed among those who were homozygous or heterozygous for the rs762551 A polymorphism, when compared to the rs762551 C individuals (standardized mean difference [SMD] = 0.40, 95% confidence interval [CI] = 0.12–0.68, *P* = .005; SMD = 0.32, 95% CI = 0.11–0.54, *P* = .003, respectively), and this was more pronounced among smokers (SMD = 0.92, 95% CI = 0.27–1.57, *P* = .005; SMD = 0.56, 95% CI = 0.22–0.90, *P* = .001, respectively).

Due to the extensive hepatic first-pass extraction of AGM following oral administration, the concentrations of the metabolites are much higher than those of the parent drug AGM .^[30]^ In particular, our patient with the rs762551 AA genotype exhibited a significantly lower level of AGM exposure compared to the rs762551 CC genotype. Therefore, liver injury with elevated liver enzymes may not be caused by the high blood concentration of AGM itself. In contrast, dihydrodiol formation indicates that AGM is bioactivated to form epoxides during its metabolism. These reactive metabolites may be closely related to AGM toxicity.

## 6 Conclusion

The underlying mechanism of AGM-induced acute liver injury remains unclear. Patients can benefit from regular liver function monitoring, which is helpful for the diagnosis of liver injury as soon as possible. As the main metabolic enzyme of AGM, the contribution of CYP1A2 genetic polymorphism to liver injury should be seriously considered.

## Acknowledgments

The authors are thankful for the assistance provided by Mr. Zhi Qiang Du for the analysis of blood drug concentrations. We thank Yuan Shen and Qin Zhou for their guidance on key issues in the review of this article.

## Author contributions

**Conceptualization:** Shushan Wang.

**Data curation:** Kankan Qu.

**Resources:** Qing Xu.

**Writing – original draft:** Shushan Wang.

**Writing – review & editing:** Jun Wang, Zhenhe Zhou.
